# Cytokine responses to LPS in reprogrammed monocytes are associated with the transcription factor PU.1

**DOI:** 10.1002/JLB.3A0421-216R

**Published:** 2022-03-13

**Authors:** Kedeye Tuerxun, Kristine Midtbö, Eva Särndahl, Egor Vorontsov, Roger Karlsson, Alexander Persson, Robert Kruse, Daniel Eklund, the X‐HiDE Consortium

**Affiliations:** ^1^ Faculty of Medicine and Health, School of Medical Sciences Örebro University Örebro Sweden; ^2^ Inflammatory Response and Infection Susceptibility Centre (iRiSC), Faculty of Medicine and Health Örebro University Örebro Sweden; ^3^ Proteomics Core Facility, Sahlgrenska Academy University of Gothenburg Sweden; ^4^ Department of Infectious Diseases, Institute of Biomedicine Sahlgrenska Academy of the University of Gothenburg Sweden; ^5^ Department of Clinical Microbiology Sahlgrenska University Hospital, Region Västra Götaland Sweden; ^6^ Nanoxis Consulting AB Gothenburg Sweden; ^7^ Department of Clinical Research Laboratory, Faculty of Medicine and Health Örebro University Örebro Sweden

**Keywords:** endotoxin tolerance, immunosuppression, inflammation, MDSCs, sepsis

## Abstract

Myeloid‐derived suppressor cells (MDSCs) are functionally immunosuppressive cells that arise and expand during extensive inflammatory conditions by increased hematopoietic output or reprogramming of immune cells. In sepsis, an increase of circulating MDSCs is associated with adverse outcomes, but unique traits that can be used to identify increased activity of MDSCs are lacking. By using endotoxin tolerance as a model of sepsis‐induced monocytic MDSCs (M‐MDSC‐like cells), this study aims to identify the mediator and transcriptional regulator profile associated with M‐MDSC activity. After analyzing 180 inflammation‐associated proteins, a profile of differentially expressed cytokines was found in M‐MDSC‐like cells versus normal monocytes stimulated with LPS. These cytokines were associated with 5 candidate transcription factors, where particularly PU.1 showed differential expression on both transcriptional and protein levels in M‐MDSC‐like cells. Furthermore, inhibition of PU.1 led to increased production of CXCL5 and CCL8 in M‐MDSC‐like cells indicating its role in regulating the ability of M‐MDSC‐like cells to recruit other immune cells. Taken together, the study identifies a unique profile in the pattern of immune mediators defining M‐MDSC activity upon LPS stimulation, which offers a functional link to their contribution to immunosuppression.

## Graphical Abstract

Differential cytokine response in endotoxin induced M‐MDSC‐like cells and their associated regulators.

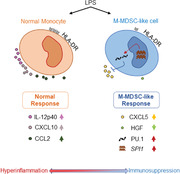

**Abbreviations**
CD14: cluster of differentiation 14
*EPAS1*
: endothelial PAS domain protein 1
G‐MDSCs: granulocytic myeloid‐derived suppressor cells
HGF: hepatocyte growth factor
*HIF1A*
: hypoxia‐inducible factor 1‐alpha
HLA‐DR: human leukocyte antigen – DR isotype
*HPRT1*
: hypoxanthine‐guanine phosphoribosyltransferase
IPA: ingenuity pathway analysis
LIF: leukemia inhibitory factor
LILRB4: leukocyte immunoglobulin‐like receptor subfamily B member 4
MDSCs: myeloid‐derived suppressor cells
M‐MDSCs: monocytic myeloid‐derived suppressor cells
*NFKBIA*
: NF‐Kappa‐B inhibitor alpha
NPX: Normalized Protein eXpression
OSM: oncostatin M
PEA: proximity extension assay
PMN‐MDSCs: polymorphonuclear myeloid‐derived suppressor cells
*PPIB*
: peptidyl‐prolyl cis‐trans isomerase B
*SIRT1*
: sirtuin 1
*SPI1*
: Spi‐1 Proto‐Oncogene
*TBP*
: TATA‐binding protein
uPA: urokinase‐type‐plasminogen‐activator
VEGFA: vascular endothelial growth factor A.

## 1 INTRODUCTION

Immunosuppressive mechanisms are essential for regulation of the immune responses in order to prevent excessive tissue damage during an inflammatory event. The cell‐mediated suppression is largely mediated through a number of specific immune cells, for example, regulatory T cells and myeloid‐derived suppressor cells (MDSCs).^1^, ^2^ Although these cells are important for immunologic homeostasis, they are also found to be involved in the pathology of diseases, like sepsis and cancer, if not effectively regulated.^3^, ^4^, ^5^

MDCSs constitute a heterogeneous population of myeloid cells with immunosuppressive function that expand during infection, inflammation, and cancer, by increased hematopoietic output or by reprogramming of immune cells.^6^, ^7^ MDSCs can be divided into 2 main subtypes: granulocytic‐MDSCs or polymorphonuclear‐MDSCs (G‐MDSCs or PMN‐MDSCs) and monocytic MDSCs (M‐MDSCs).^8^ The reprogramming of monocytes into M‐MDSC is initiated by exposure to damage‐associated molecular patterns and pathogen‐associated molecular patterns.^9^ In the case of LPS‐mediated reprogramming, this phenomenon is also known as endotoxin tolerance.^4^, ^6^, ^7^, ^10^ The reprogrammed immunosuppressive monocytes in experimental models, referred to as M‐MDSC‐like cells,^6^ display similar properties as M‐MDSCs studied in cancer^6^ and sepsis,^11^, ^12^ including a drastic reduction of proinflammatory TNF production and an impaired antigen presenting capacity. The latter is indicated by decreased surface expression of MHC class II molecules, such as HLA‐DR.^10^, ^13^

MDSCs were first discovered as “suppressor cells” in tumors^14^ but recently MDSCs have been investigated in other pathologic conditions, including sepsis,^5^ trauma,^15^ and most recently in COVID‐19.^16^ In sepsis, a correlation between low HLA‐DR expression and impaired TNF production in septic monocytes upon LPS stimulation has been demonstrated,^17^ and this endotoxin tolerance has been suggested to be associated with sepsis severity.^12^ Immunosuppression further contributes to mortality, in particular at later stages of sepsis, where the numbers of MDSCs in the circulation increase.^11^, ^18^ Although MDSCs have gained attention as critical regulators of the inflammatory response during sepsis, there is still a lack of unique traits that can be used to identify increased activity of MDSCs, and to potentially identify septic patients in an early phase of immunosuppression. The aim of this study was to identify mediators, associated with M‐MDSC activity and their potential transcriptional regulators upon LPS stimulation, to provide a profile specific for M‐MDSC identification.

## 2 MATERIALS AND METHODS

### 2.1 Cell culture

Primary human monocytes were isolated from buffy coats collected from anonymous healthy blood donors by the Department of Transfusion Medicine at Örebro University Hospital, Sweden. The study was conducted in compliance with the ethical guidelines of Helsinki as well as the ethical policy at Örebro University Hospital. All donors had signed a consent allowing the blood to be used for research purposes. The blood samples were anonymized by the Department of Transfusion Medicine at Örebro University Hospital, and no personal information can thereby be tracked back. As the buffy coats were prepared in connection to a regular blood donation, the donors were not exposed to any additional harm or risk. According to the paragraph 4 of Swedish law (2003:460), this study did not require ethical approval.

The PBMCs were separated by density gradient centrifugation on Lymphoprep (Axis‐Shield, Oslo, Norway) and the CD14^+^ cells were isolated by magnetic sorting using CD14 MACS microbeads (Miltenyi Biotech, Bergisch Gladbach, Germany) according to manufacturer's protocol. The cells were cultured in RPMI (Gibco, Thermo Fisher Scientific, Waltham, MA; cat. nr:31870‐025) or DMEM (BioWhittaker, Lonza, Basel, Switzerland; BE12‐733F) supplemented with 10% human AB+ serum (pooled from 5 healthy blood donors collected under the same conditions as buffy coats), l‐glutamine (10 mM), sodium pyruvate (10 mM), and glucose (4.5 g/L) (all from Gibco, Thermo Fisher Scientific).

### 2.2 Generation of M‐MDSC‐like cells and LPS challenge

**FIGURE 1:**
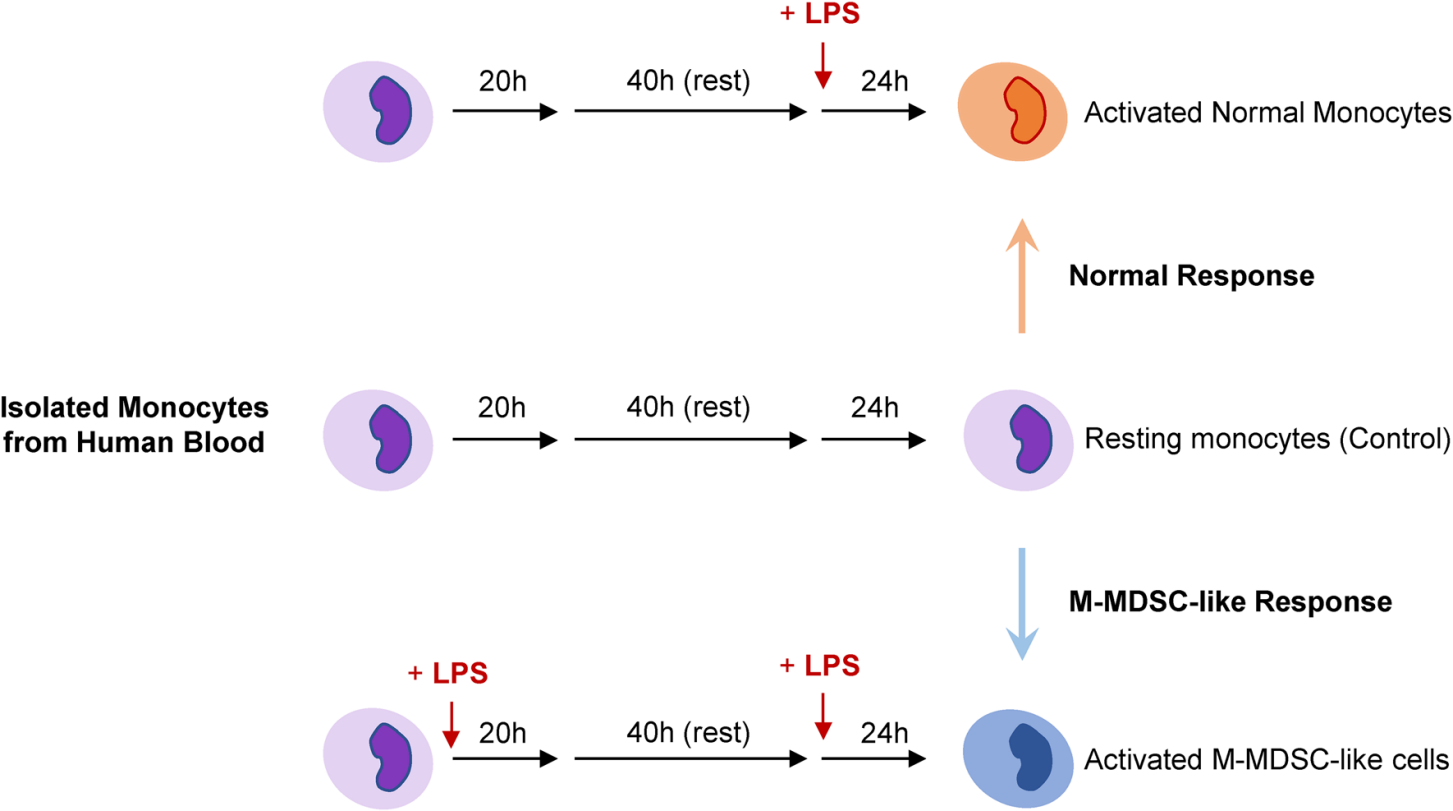
**A representation of used cell model**. Isolated primary CD14^+^ cells were seeded (1.5 × 10^6^/cm^2^) into culture flasks, either left untreated (monocytes) or stimulated with LPS (10 ng/ml) for 20 h (M‐MDSC‐like cells). Following a medium change and a 40 h rest, the cells were reseeded and subjected to an initial challenge with LPS (10 ng/ml, normal cells) or rechallenged with LPS (10 ng/ml, M‐MDSC‐like cells)

To induce M‐MDSC‐like cells, the isolated primary monocytes from each donor were divided into 2 fractions and seeded (1.5 × 10^6^/cm^2^) in culture flasks (Sarstedt, Nümbrecht, Germany), either stimulated with LPS (LPS‐B5 (E. coli serotype 055: K59(B5)H‐), 10 ng/ml Invivogen, San Diego, CA) for 20 h (M‐MDSC‐like cells) or left untreated (normal monocytes). Other stimuli used, such as recombinant human IL‐1β (*E. coli* expressed human IL‐1β with HSA), Pam2CSK4 (synthetic diacylated lipoprotein), and Pam3CSK4 (synthetic triacylated lipopeptide), were also purchased from Invivogen. Following a medium change, both fractions of cells were then allowed to rest for 40 h. Following the resting period, the cells were reseeded and were challenged with 10 ng/ml LPS. A schematic presentation of the timeline can be seen in Figure 1.

### 2.3 Flow cytometry

Pelleted cells were resuspended in 1× Annexin V binding buffer and stained with PE‐Annexin V and 7AAD (BioLegend, San Diego, CA) together with FITC mouse anti‐human HLA‐DR (Clone G46‐6; BD Biosciences, Franklin Lakes, NJ) for 30 min. A FITC mouse IgG2a isotype control (BD Biosciences) was used for all experiments. The data were collected on a Accuri C6 (BD Biosciences) and analyzed using the Kaluza software (Beckman Coulter, Brea, CA).

### 2.4 Olink Proteomics

A total of 180 immune mediators were analyzed in cell culture supernatants from 4 individual donors using a proximity extension assay (PEA, Olink Proteomics, Uppsala, Sweden) coupling oligonucleotide‐tagged antibodies to a qPCR reaction, from which the amplified sequences were quantified.^19^ The arrays used were “immune response” and “inflammation,” as available from the manufacturer. Processing, output data, quality check, and normalization were performed by Olink Proteomics. Data were obtained as Normalized Protein eXpression (NPX) values on a log2 scale. Eighteen mediators, below detection limit, were excluded from further analysis. The molecules that showed the strongest association to M‐MDSC‐like cells or normal monocytes compared with unstimulated monocyte control were selected for further quantification.

### 2.5 Quantification of immune mediators

Cells of each phenotype were seeded, at 5 × 10^5^cells in 1 ml medium in a 24‐well cell culture plate for each experiment and cell culture supernatants were collected at indicated time points, where cells and debris were eliminated by centrifugation prior to measurements. TNF concentrations were analyzed using ELISA (BioLegend) according to the manufacturer's instructions. For the validation of selected molecules identified through Olink proteomics, new experiments as delineated above were repeated and cell culture supernatants were collected from 5 new, individual experiments. Concentrations (lower limit of detection) of IL‐1α (0.3 pg/ml), IL‐10 (0.04 pg/ml), IL‐12/IL‐23p40 (0.9 pg/ml), TNF (0.4 pg/ml), VEGF‐A (2.9 pg/ml), CCL2 (2.4 pg/ml), CCL4 (1.9 pg/ml), CCL8 (0.06 pg/ml), CXCL5 (0.3 pg/ml), and CXCL10 (0.4 pg/ml) were measured using a customized U‐Plex kit (Meso Scale Discovery, Rockville, MD) detected by electrochemiluminescence in Meso QuickPlex SQ 120 (Meso Scale Discovery). Quantification of CXCL‐6 (34.3 pg/ml), HGF (15.1 pg/ml), LIF (30.3 pg/ml), TGF‐α (6.8 pg/ml), and uPA (116.6 pg/ml) was performed by Human Magnetic Luminex Assay, a multiplex bead technology (R&D systems, Inc. Minneapolis, MN). The samples were analyzed on a Luminex®200™ instrument (Invitrogen, Merelbeke, Belgium), and the data were collected using the xPONENT 3.1™ (Luminex Corporation, Austin, TX).

All analyses were performed according to manufacturer's instructions. Samples with a coefficient of variation (CV) > 20% were excluded.

### 2.6 Ingenuity pathway analysis

Molecular NPX data obtained from PEA as well as data from the validation experiments were uploaded into Ingenuity Pathway Analysis 2.4 software (Ingenuity Systems, www.ingenuity.com), for analysis of potential upstream transcription regulators with the Ingenuity Knowledge Base.

### 2.7 Extraction of mRNA, conversion to cDNA and qPCR

Two hours after LPS challenge, cells from each phenotype were collected and lysed in Buffer RLT (Qiagen, Hilden, Germany). Total RNA from lysates derived from 5 × 10^5^ cells was extracted using RNeasy Mini Kit (Qiagen) according to manufacturer's instructions, reusing solution in the final elution step to maximize RNA concentration. RNA quantity was measured with NanoDrop 2000 (Thermo Fisher Scientific). The ratio of absorbance at 260 and 280 nm from NanoDrop at approximately 1.5‐2.0 was accepted as pure. cDNA was synthesized in a 40 μl reaction containing 500 ng RNA, using the High‐Capacity cDNA Reverse Transcription Kit (Applied Biosystems, Foster City, CA) in a LifePro Thermal Cycler (Bioer, Hangzhou, China).

Real‐time PCR was performed in a Quantstudio 7 Flex Real‐Time PCR system (Applied Biosystems). Sample cDNA (2 μl) was added to TaqMan Fast Universal PCR Master Mix (Applied Biosystems) and TaqMan Gene Expression Assays (Applied Biosystems; see Table 1 for details), according to the manufacturer's instructions to achieve a final reaction volume of 10 μl. Water was included as a negative control in every run to check for cross contamination. Pipetting of samples and reaction mixtures into 384‐well plate was performed by PIRO Pipetting Robot (Dornier, Lindau, Germany). The PCR protocol started with an initial denaturation phase at 95°C for 20 s, followed by 40 amplification cycles at 95°C for 1 s, 60°C for 20 s. Peptidyl‐prolyl cis‐trans isomerase B (PPIB) was determined as reference gene by using NormFinder R package (MOMA, Aarhus University Hospital, Denmark) for normalization among a total of 3 candidate reference genes. For cDNA quantification, a 6‐point serially 4‐fold diluted calibration curve was developed from PBMCs stimulated by 1 μg/ml LPS and cultured with RPMI complete cell culture medium. The RNA of treated PBMCs was extracted from 1 × 10^7^ cells per reaction using QIAamp RNA Blood Mini Kit (Qiagen). cDNA reverse transcription was conducted by the same procedure as sample cDNA synthesis.

**TABLE 1:** Description of assays in real‐time PCR

Gene	TaqMan assay ID	Assay
*HPRT1*	Hs02800695_m1	Reference gene
*TBP*	Hs00427620_m1	Reference gene
*PPIB*	Hs00168719_m1	Reference gene
*SPI1*	Hs02786711_m1	Target gene
*HIF1A*	Hs00153153_m1	Target gene
*NFKBIA*	Hs00355671_g1	Target gene
*SIRT1*	Hs01009006_m1	Target gene
*EPAS1*	Hs01026149_m1	Target gene

*HPRT1*, hypoxanthine‐guanine phosphoribosyltransferase; *TBP*, TATA‐binding protein; *PPIB*, peptidyl‐prolyl cis‐trans isomerase B; *SPI1*, Spi‐1 proto‐oncogene; *HIF1A*, hypoxia‐inducible factor 1‐alpha; *NFKBIA*, NF‐Kappa‐B inhibitor alpha; *SIRT1*, Sirtuin 1; *EPAS1*, endothelial PAS domain protein 1.

All samples were amplified in duplicate and the mean quantity values were obtained for further data analysis. The threshold for the CV value between duplicates was set to <0.15. The samples with higher CV values were rerun. Cycle threshold (CT) cut‐off value was set to 35. All reactions had an efficiency between 89% and 110%, which corresponds to a slope between −3.63 and −3.10.

### 2.8 Liquid chromatography–mass spectrometry‐based proteomics

Twenty‐four hours after LPS challenge, 1 × 10^6^ cells from each phenotype were pelleted and flash‐frozen in liquid nitrogen and stored in −80°C before preparation for liquid chromatography–mass spectrometry (LC–MS) analysis (see detailed description of the proteomic sample preparation, LC–MS methods, and data processing in the Supplementary Document 1). In brief, the samples were homogenized using a FastPrep®−24 instrument (MP Biomedicals, Irvine, CA), and aliquots containing 30 μg of total protein material from each sample were processed using the modified filter‐aided sample preparation method.^20^ The protocol included digestion with trypsin (Pierce MS Grade, Thermo Fisher Scientific) in the buffer containing 0.5% sodium deoxycholate and 50 mM triethylammonium bicarbonate, labeling with Tandem Mass Tag (TMT 10plex) reagents (Thermo Fischer Scientific) according to the manufacturer's instructions, desalting, and fractionation by basic‐pH reversed‐phase chromatography (bRP‐LC). The primary fractions were concatenated into final 20 fractions (1+21, 2+22, … 20+40), evaporated and reconstituted in 15 μl of 3% acetonitrile with 0.2% formic acid.

The fractions were analyzed on an Orbitrap Fusion Lumos Tribrid mass spectrometer interfaced with an Easy‐nLC 1200 liquid chromatography system (both Thermo Fisher Scientific). Precursor ion spectra were recorded at 120,000 target resolution, most abundant precursors were fragmented by collision‐induced dissociation at 35% collision energy, the TMT reporter ions were generated by higher‐energy collision dissociation at 65% collision energy and the MS3 spectra were recorded at 50,000 resolution. Targeted inclusion list was prepared for the selected peptides of the proteins P25963 (NF‐kappa‐B inhibitor alpha, NFKBIα), Q16665 (hypoxia‐inducible factor 1‐alpha, HIF‐1α), and Q99814 (endothelial PAS domain‐containing protein 1, EPAS‐1) that were detectable according to the data in ProteomicsDB (https://www.ProteomicsDB.org).^21^ The fractions were reanalyzed using the modified LC–MS method that only fragmented the precursor ions with the correct charge and the monoisotopic mass within 15 ppm of the theoretical mass in the inclusion list.

Proteins were identified and quantified using Proteome Discoverer version 2.4 (Thermo Fisher Scientific). The database matching was performed using the Mascot search engine v. 2.5.1 (Matrix Science, London, UK) against the Swiss‐Prot *Homo sapiens* database. Percolator was used for peptide‐spectrum match (PSM) validation with the strict false discovery rate (FDR) threshold of 1%. The LC–MS files from the reinjection experiment were matched against the protein database that consisted only of the proteins P25963 (NF‐kappa‐B inhibitor alpha), Q16665 (HIF‐1α) and Q99814 (EPAS‐1), and the Fixed Value PSM Validator was used instead of Percolator.

### 2.9 Inhibition of transcription factor PU.1

M‐MDSC‐like cells were treated by pharmacologic inhibitor of PU.1, DB1976^22^, ^23^ (MCE, New Jersey, USA) in 9 individual experiments. The concentration of the inhibitor was decided by titration experiments, where 5 × 10^5^ cells were seeded into a 24‐well plate, treated with 5 nM, 50 nM, 0.5 μM, 5 μM, 50 μM of DB1976 or DMSO as vehicle control and an unstimulated monocyte control. Cell viability was evaluated using flowcytometry, as described earlier, 24 h after treatment (data not shown).

1 × 10^5^ cells of each phenotype were seeded in 96‐well plates in 200 μl medium, where M‐MDSC‐like cells were treated with 5 μM DB1976 or 0.5% DMSO as vehicle control 1 h before challenged by the second dose of LPS. In each experiment, M‐MDSC‐like cells stimulated with second LPS (M‐MDSC‐like response), monocytes treated with first LPS (normal monocyte responses), and unstimulated monocyte controls were included in parallel with PU.1 inhibition. At 24 h of the treatment, supernatants were analyzed for selected molecules using a customized U‐Plex kit (Meso Scale Discovery) detected by electrochemiluminescence in Meso QuickPlex SQ 120 (Meso Scale Discovery) as described above.

### 2.10 Statistical analysis

In the initial analysis of 180 immune mediators, each set of phenotype data was compared with control (unstimulated monocytes) using Student's *t*‐test, followed by FDR correction (Benjamini–Hochberg). Initially, a low stringency (*p* = 0.15) was used to allow identification of a larger number of potential candidates that could be selected for subsequent validation experiments. Comparisons of cytokines in the following validation and inhibition experiments as well as gene expression levels between phenotypes and control were analyzed using repeated measures ANOVA, followed by a Holm–Sidak's posthoc test for between‐group comparisons. The absolute levels of cytokines were log transformed to obtain a normal data distribution prior to statistical analysis. For comparisons of HLA‐DR surface expression and transcription factor protein abundance between M‐MDSC‐like cells and normal monocytes, a paired *t*‐test was used. For the proteomics data, it should be noted that >7000 proteins were identified, but only the abundance of the selected transcription factors was used for the preplanned, down‐stream statistical analyses of identified transcription factors. All analyses were done in GraphPad Prism v. 7.04.

## 3 RESULTS

### 3.1 Exploring the differences in LPS response

**FIGURE 2:**
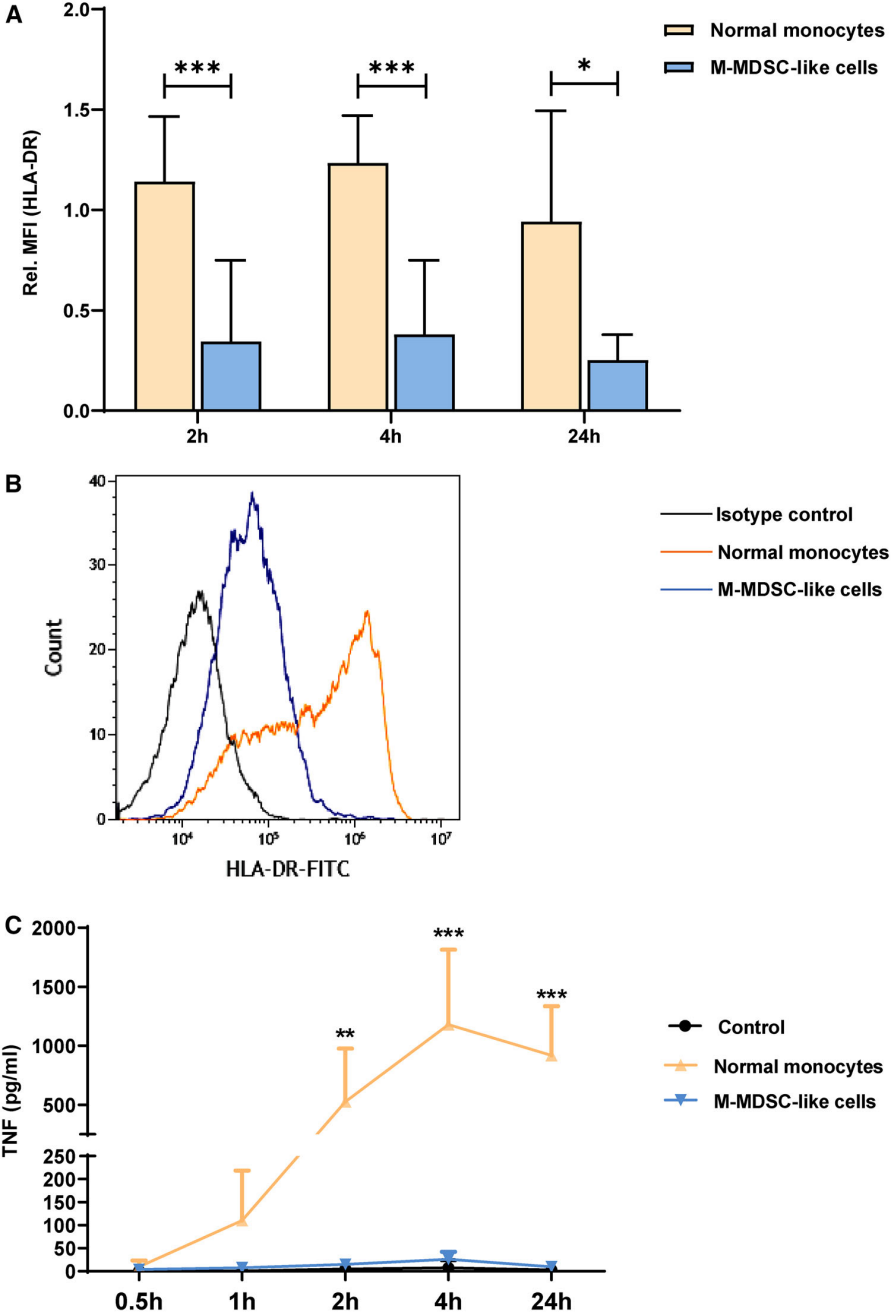
**Surface expression of HLA‐DR and TNF levels in supernatants**. (A) Surface expression of HLA‐DR and (B) A representative histogram showing the HLA‐DR expression in monocytes and M‐MDSC‐like cells. (C) TNF levels were determined in untreated monocytes (control) and LPS‐challenged normal monocytes (normal monocytes), as well as LPS‐challenged M‐MDSC‐like cells (M‐MDSC‐like cells) at the indicated time points (*n* = 5). The MFI of HLA‐DR for each phenotype was normalized to the MFI of HLA‐DR in untreated cells. Statistical analysis was performed using paired *t* test (HLA‐DR) and repeated measures ANOVA with a Holm–Sidak's posthoc test (TNF). Data are log transformed, shown as mean and standard deviation. Values of *p* < 0.05 (*), *p* < 0.01(**), *p* < 0.001(***) were considered significant

The confirmation of the M‐MDCS‐like phenotype was evaluated by TNF quantification using ELISA as well as measurement of cell surface HLA‐DR expression by flow cytometry. The HLA‐DR expression was significantly lower in M‐MDSC‐like cells compared with normal monocytes (Figures 2(A) and 2(B)). TNF production in M‐MDSC‐like cells at 2, 4, and 24 h after LPS challenge were significantly lower compared with normal monocytes, where no difference was observed compared to unstimulated monocyte control (Figure 2(C)). The TNF level was not affected by increasing the LPS concentration (from 10 to 100 ng/ml or 1000 ng/ml) nor by addition of other stimuli, such as TLR ligands Pam2CSK4 (10 ng/ml), Pam3CSK4 (10 ng/ml), or recombinant IL‐1β (500 pg/ml) (Figure S1). In addition, unstimulated M‐MDSC‐like cells showed decreased HLA‐DR expression compared with normal monocytes, and with no spontaneous TNF production (data not shown). Furthermore, at 24 h poststimulation, the recovery rate of the harvested cells was 43% in normal monocytes and 30% in M‐MDSC‐like cells and approximately 80% of the cells remained viable, with no significant differences between the 2 phenotypes (data not shown).

To explore the difference in response associated with the 2 phenotypes (M‐MDSC‐like cells and normal monocytes, respectively), a set of 180 immune mediators, using PEA, was measured in LPS stimulated M‐MDSC‐like cells and normal monocytes as well as unstimulated monocyte controls, at 24 h poststimulation. Among the 180 unique immune proteins analyzed, 18 were not detectable in any of the conditions. For the remaining proteins, when compared with the unstimulated monocyte controls, a total of 23 mediators showed differences in either M‐MDSC‐like cells, normal monocytes, or both. To allow a larger set of proteins to be included in the following validation experiments, the selection was performed using a low stringent FDR corrected *p* value (*p* ≤ 0.15). Depending on if the differences could be seen in both or only 1 phenotype, these molecules were divided into 3 groups; 4 molecules showed a difference at the designated *p* value only in normal monocytes (normal response candidates), 8 molecules only in M‐MDSC‐like cells (M‐MDSC‐like response candidates), and 11 molecules were affected in both phenotypes (shared response candidates) (Table 2). Within the shared response candidates, 4 mediators (CCL2, CCL4, IL‐10, TNF) showed a difference at the designated *p* value in terms of quantified levels between the M‐MDSC‐like cells and normal monocytes.

**TABLE 2:** Different responses from endotoxin‐induced M‐MDSC‐like and normal monocytes compared with control

	Normal monocytes versus Ctrl		M‐MDSC‐like cells versus Ctrl		
Analytes	Benji‐Hoch corrected *p* values	Fold change	Benji‐Hoch corrected *p* values	Fold change	Response group
CXCL5	0.2207	3.6839	0.0601	3.5022	M‐MDSC‐like response
CXCL6	0.1882	4.0317	0.0027	4.4178	M‐MDSC‐like response
HGF	0.5579	−0.6067	0.0361	−2.204	M‐MDSC‐like response
IL‐1α	0.2678	3.1270	0.0027	5.9488	M‐MDSC‐like response
LILRB4	0.1882	1.9644	0.1149	2.0595	M‐MDSC‐like response
TGF‐α	0.1666	2.8625	0.0601	2.084	M‐MDSC‐like response
uPA	0.9506	−0.0981	0.0788	0.828	M‐MDSC‐like response
VEGFA	0.1666	2.5831	0.1098	1.1664	M‐MDSC‐like response
CCL8	0.0054	9.5912	0.2749	6.0375	Normal response
CXCL10	0.1345	7.5705	0.7356	0.6841	Normal response
IL‐12p40	0.0835	3.8653	0.6579	0.2095	Normal response
LIF	0.1345	1.6865	0.2054	1.6604	Normal response
CCL20	0.0054	8.4055	0.0027	8.4558	Shared
CCL3	0.0993	6.8014	0.0777	6.8255	Shared
CCL7	0.0835	7.3291	0.0119	6.2424	Shared
CXCL1	0.0835	5.7601	0.0027	5.9299	Shared
IL‐6	0.0054	12.0924	0.0027	12.0898	Shared
IL‐8	0.1345	4.1649	0.0109	4.6673	Shared
OSM	0.0993	2.3119	0.0312	2.7793	Shared
CCL2	0.1345	4.7352	0.0119	5.5584	Shared^a^
CCL4	0.0835	8.4159	0.0047	7.7838	Shared^a^
IL‐10	0.0815	8.6616	0.0027	6.2716	Shared^a^
TNF	0.0993	12.2397	0.0109	1.1655	Shared^a^

A total of 180 unique immune markers were analyzed using proximity‐extension assay (PEA) in supernatants from untreated monocytes (control), LPS‐challenged monocytes (normal), and LPS‐challenged M‐MDSC‐like cells (MDSC‐like). The table shows the Benji‐Hoc *p* values and the level of molecules in normal monocytes (A) and M‐MDSC‐like cells (B) when compared with control (*n* = 4). The molecules were divided into 3 group depending on if the differences (*p* ≤ 0.15) could be seen in both or only 1 phenotype; as in normal response, M‐MDSC‐like cell response and shared response.

^a^ Molecules that showed a significant difference between the normal monocytes and M‐MDSC‐like cells.

### 3.2 Validation of the phenotypic responses

**FIGURE 3:**
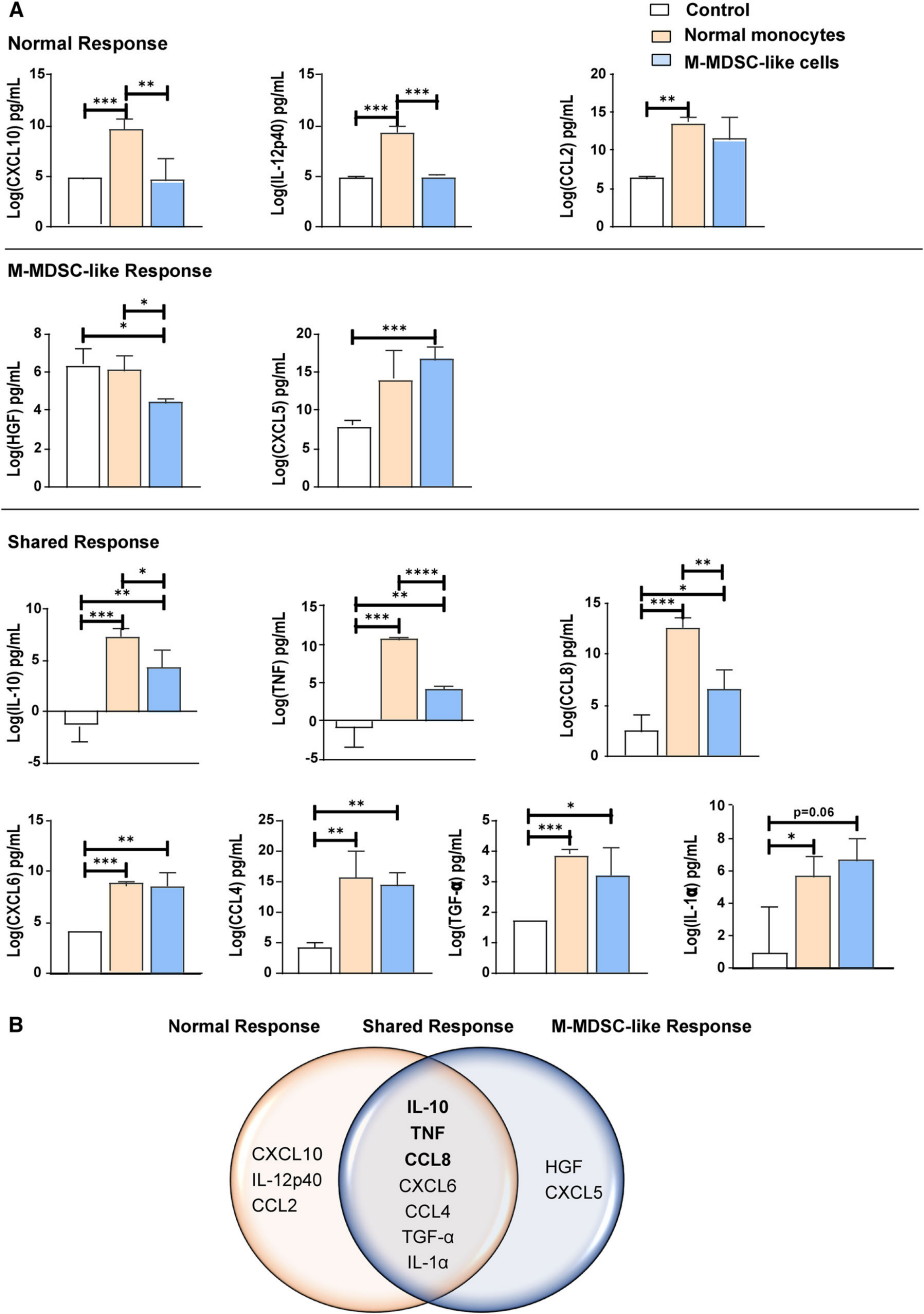
**Validation of the response profiles**. (A) The concentration of the immune mediators upon LPS stimulation was determined in supernatants from controls, normal monocytes, and M‐MDSC‐like cells (*n* = 5). (B) Mediators that showed significant difference when compared with control were divided into normal response (orange), M‐MDSC‐like cell response (blue), or shared response (cross section) in the Venn‐diagram. The mediators that also showed significant differences between the 2 phenotypes are bolded. Statistical analysis was performed using repeated measures ANOVA, followed by a Holm–Sidak's posthoc test. Data are log2 transformed, shown as mean and standard deviation. Values of *p* < 0.05 (*), *p* < 0.01(**), *p* < 0.001(***) were considered significant

Following PEA analysis, we performed further validation experiments using absolute quantitative methods, namely customized U‐Plex kit detected by electrochemiluminescence in Meso QuickPlex SQ 120 (Meso Scale Discovery) and the Human Magnetic Luminex Assay (R&D systems), as well as a more stringent statistical approach. The validation was done on the following 16 selected molecules derived from the previous PEA analysis, that is, the normal response candidates (CCL8, CXCL10, IL‐12p40, and LIF), the M‐MDSC‐like response candidates (HGF, IL‐1α, CXCL5, CXCL6, LILRB4, VEGF‐A, TGF‐α, and uPA), and the 4 shared response candidates that showed a significant difference between the phenotypes (CCL2, CCL4, IL‐10, and TNF). The result of the validation experiments showed that among the above mentioned 16 selected candidates, significant differences were seen in 12 mediators, when comparing each phenotype to unstimulated control (Figure 3(A)). These 12 mediators were regrouped, based on the validation experiments, as: normal response (CXCL10, IL‐12p40, and CCL2), M‐MDSC‐like response (HGF and CXCL5), and shared response (CCL8, IL‐10, TNF, CXCL6, CCL4, TGF‐α, and IL‐1α) (Figure 3(B)), where TNF, IL‐10, and CCL8 also showed a significant difference between the 2 phenotypes (Figure 3A). The remaining 4 mediators could not be quantified using electrochemiluminescence or cytometric bead assays.

### 3.3 Changes in gene expression and relative protein abundance of potential upstream regulators

**FIGURE 4:**
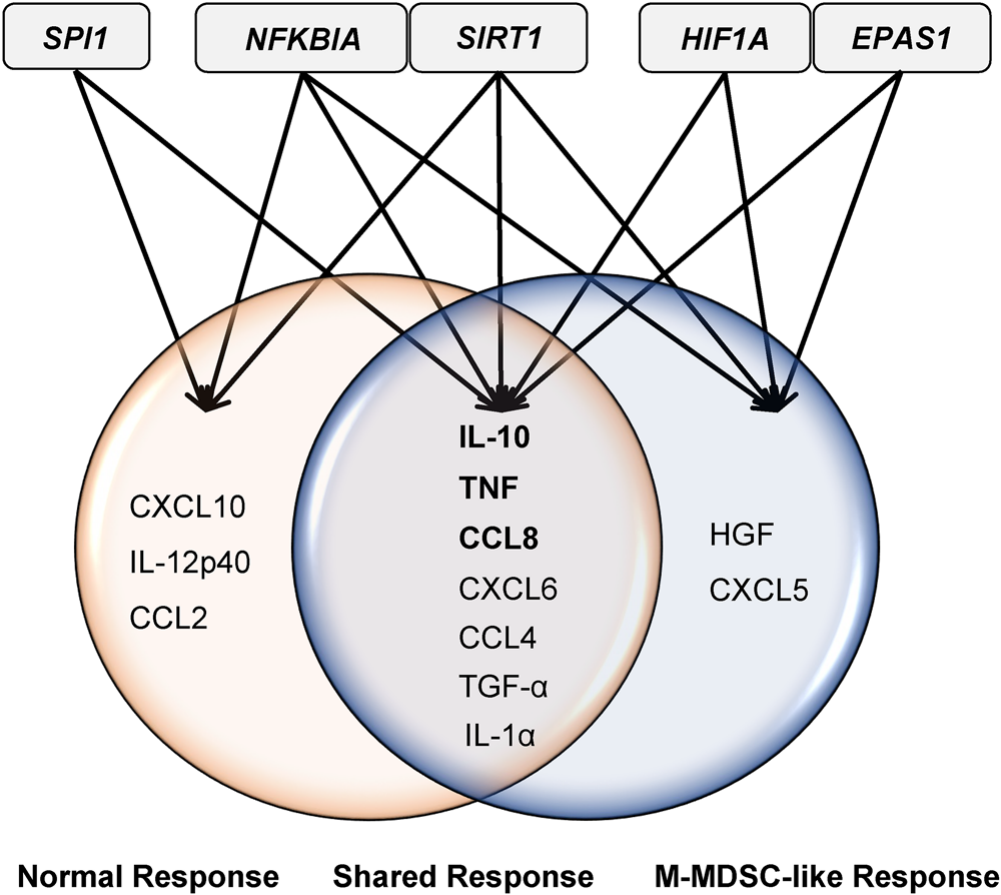
The connections of the transcription factors with the downstream response mediators. Data obtained by the PEA (Table 2) as well as the response panel (Figure 3) were used to curate potential transcription factors candidates. Five transcription factors, *HIF1A*, *SPI1*, *EPAS1*, *NFKBIA*, and *SIRT1*, were selected for further evaluation in qPCR. The arrows represent interactions of transcription factors and the mediators discovered in IPA

The potential upstream regulators governing the identified differences in the responses between the 2 phenotypes were explored using IPA. The data set obtained from PEA as well as further validation experiments was uploaded into the IPA software, and was used to curate a list of potential upstream transcription factor candidates. These candidates were further filtered based on their connections with both of the “phenotype specific” and “shared” response mediators, based on the software's literature‐based findings. With further literature study, 5 transcription factors, *HIF1A*, *SPI1*, *EPAS1*, *NFKBIA*, and *SIRT1*, which all had connections with both shared and phenotype‐specific mediators, were selected for further evaluation by qPCR and mass spectrometry‐based proteomics. A schematic illustration over the connections between the transcription factors and the different phenotypic responses are shown in Figure 4.

**FIGURE 5:**
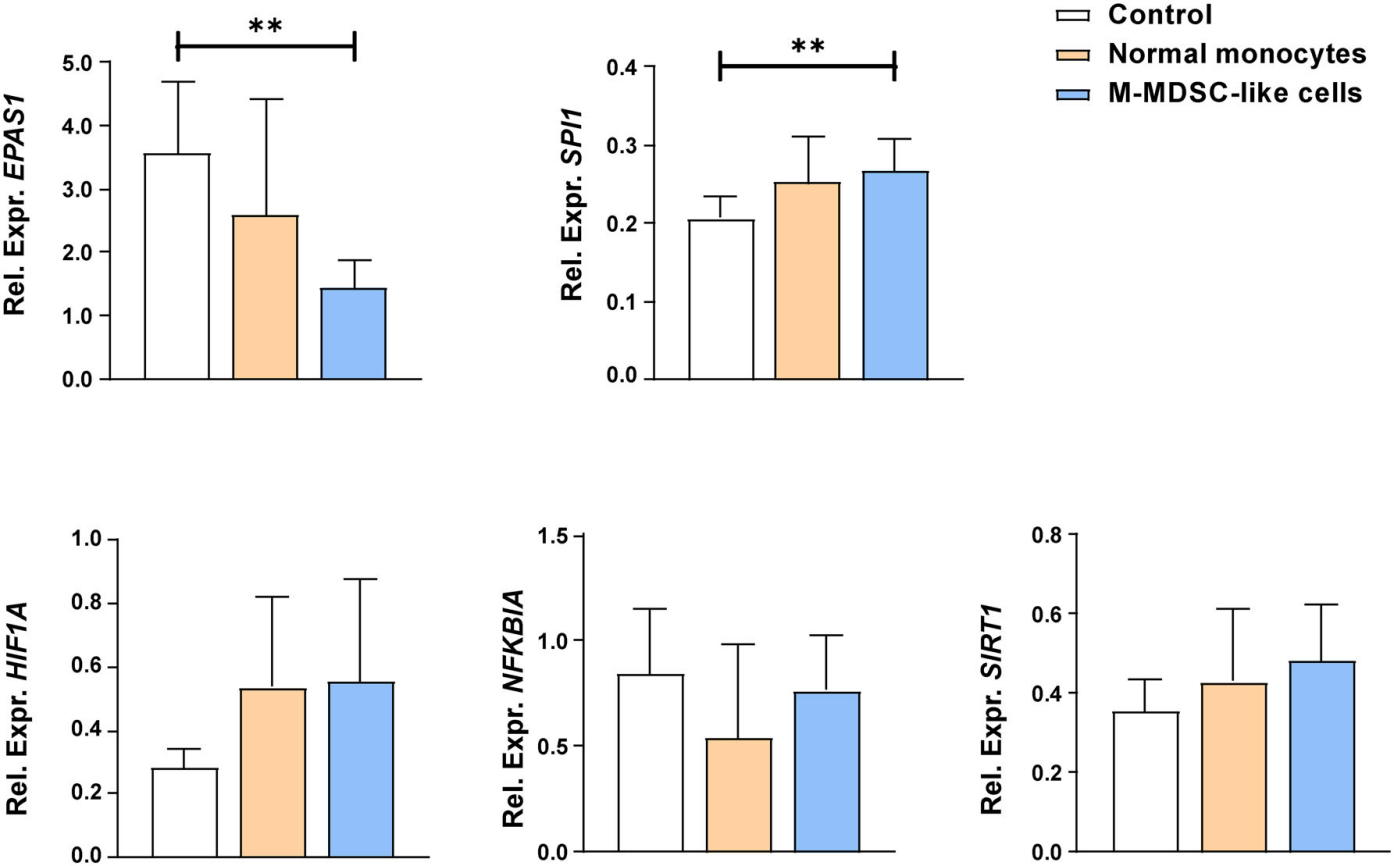
**Expression of selected transcription factors**. Total RNA was extracted from cells at 2 h after the second LPS challenge (*n* = 7). The relative expression level of the transcriptional factors *EPAS1*, *SPI1*, *HIF1A*, *NFKBIA*, and *SIRT1* in normal monocytes and M‐MDSC‐like cells was compared with the untreated monocytes (control). Gene expression is normalized against *PPIB* expression. Statistical analysis was performed using repeated measures ANOVA with a Holm–Sidak's posthoc test. Data are shown as mean and standard deviation. Values of *p* < 0.05 (*), *p* < 0.01(**), *p* < 0.001(***) were considered significant

The expression levels of the transcription factors *HIF1A*, *SPI1*, *EPAS1*, *NFKBIA*, and *SIRT1*, in the 2 phenotypes at 2 h after the LPS challenge were compared with the unstimulated monocyte control. The result showed that M‐MDSC‐like cells have a decreased expression of *EPAS1* (*p* = 0.0091) and increased expression of *SPI1* (*p* = 0.0023). No significant differences in gene expression were found in *HIF1A*, *SRT1*, and *NFKBIA* between the phenotypes and unstimulated monocyte control (Figure 5).

**FIGURE 6:**
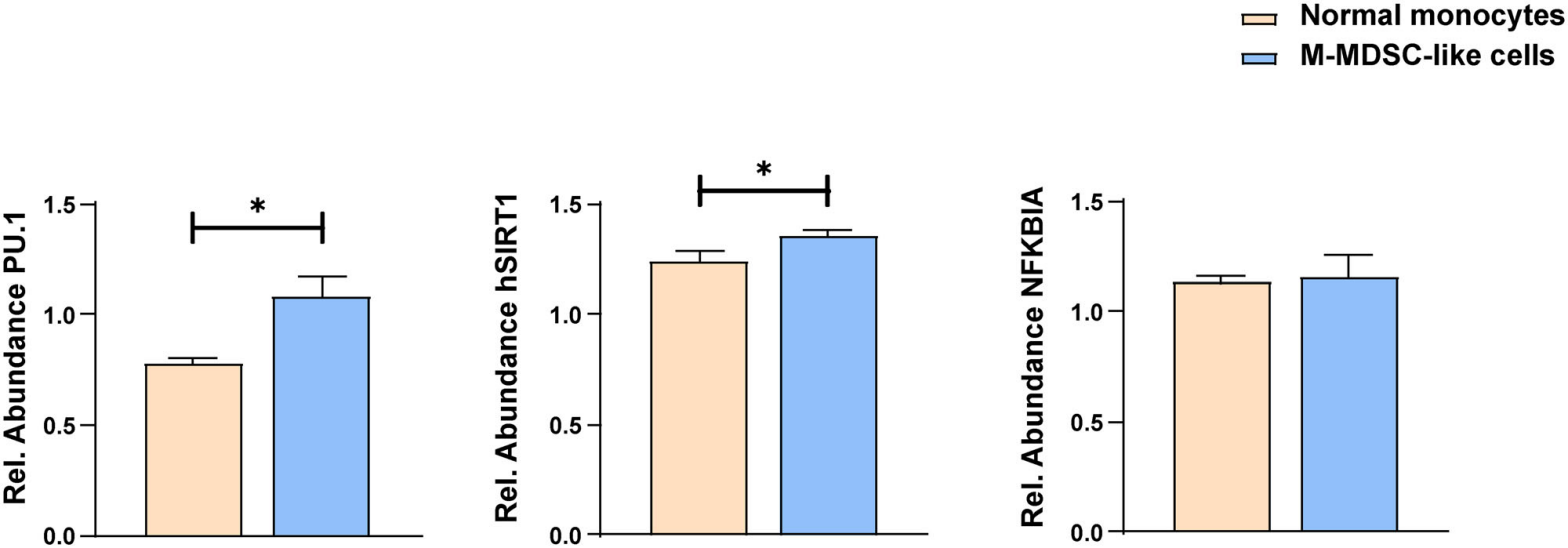
**Protein abundance of the selected transcription factors**. The abundance of selected transcription factors was investigated in normal monocytes and M‐MDSC‐like cells 24 h after LPS challenge from 1 × 10^6^ cells of each phenotype, using LC–MS (*n* = 3). Protein abundance is normalized against the value of the control. Statistical analysis was performed using paired *t* test. Data are shown as mean and standard deviation. Values of *p* < 0.05 (*), *p* < 0.01(**), *p* < 0.001(***) were considered significant

To analyze if these changes in gene expression levels of transcription factors translated into differences in protein level, the abundance of the selected transcription factor proteins 24 h after LPS challenge was compared between M‐MDSC‐like cells and normal monocytes using bottom‐up LC–MS/MS‐based proteomics. Consistent with the gene expression analysis, PU box binding‐1 (PU.1, encoded by *SPI1*) increased by 40% in relative protein abundance in M‐MDSC‐like cells. In addition to PU.1, NAD‐dependent protein deacetylase sirtuin‐1 (hSIRT1, encoded by *SIRT1*) also showed an approximate 10% increase in the relative abundance (Figure 6). Attempting to detect the other selected transcription factors, additional targeted MS experiments were performed, making use of an inclusion list containing precise *m/z* and *z* values of selected peptides from NFKBIA (encoded by *NFKBIA*), EPAS‐1, and HIF‐1α (encoded by *HIF1A*). NFKBIA was detected with 1 peptide but did not show any significant difference in relative abundance across the samples. EPAS‐1 and HIF‐1α could not be detected in any of the LC–MS experiments.

Taken together, M‐MDSC‐like cells showed an up‐regulation of the transcription factors PU.1 (both gene expression level and in protein abundance) and hSIRT1 (only protein abundance), whereas gene expression of *EPAS1* showed a down‐regulation. However, EPAS‐1 could not be confirmed on the protein level.

### 3.4 Inhibition of transcription factor PU.1

**FIGURE 7:**
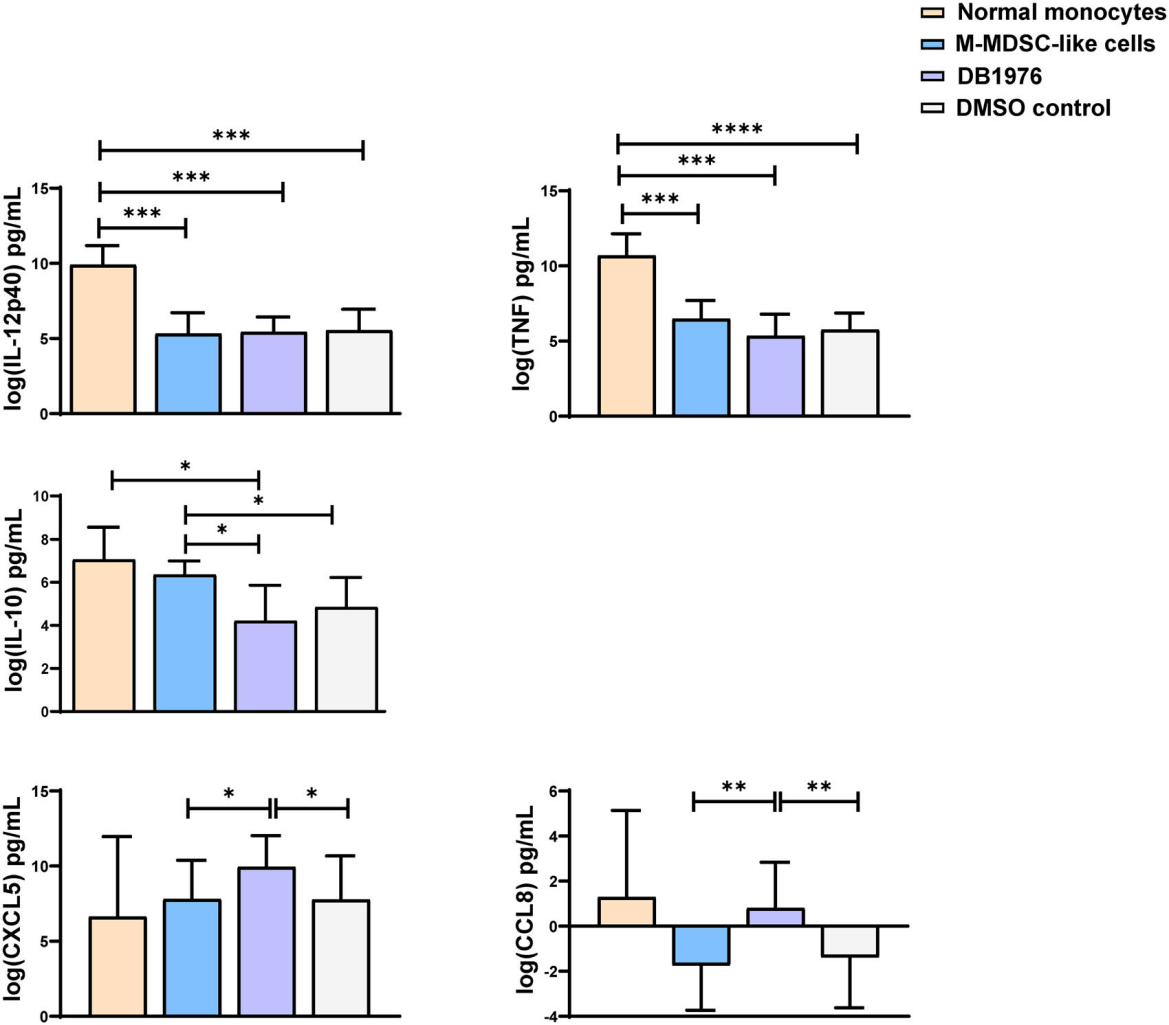
**Cytokine response of M‐MDSC‐like cells in PU.1 inhibition**. The concentration of the immune mediators upon LPS stimulation were determined in supernatants from M‐MDSC‐like cells with or without 5 μM PU.1 inhibition and normal monocytes as well as supernatants from unstimulated controls (*n* = 9). Statistical analysis was performed using repeated measures ANOVA, followed by FDR and a Holm–Sidak's posthoc test. Data are log2 transformed, shown as mean and standard deviation. Values of *p* < 0.05 (*), *p* < 0.01(**), *p* < 0.001(***) were considered significant

Since PU.1 was the transcription factor that showed a consistent increase in both gene transcription and protein levels in M‐MDSC‐like cells, its role in the response pattern was evaluated. Thus, M‐MDSC‐like cells were treated with 5 μM of the PU.1 inhibitor, DB1976^22^, ^23^ prior to LPS challenge, and selected mediators were analyzed at 24 h of treatment, including the M‐MDSC‐like response mediator CXCL5, the normal response mediator IL‐12p40, as well as TNF, IL‐10, and CCL8 of the shared response. Analysis was done by using electrochemiluminescence in the Meso QuickPlex SQ 120. The levels of the mediators were compared to the molecules released by M‐MDSC‐like cells without inhibitor. As shown in Figure 7, the differences between MDSC‐like cells and normal monocytes were as previously found (Figure 3). However, when MDSC‐like cells were treated with the PU.1 inhibitor, the levels of CXCL5 and CCL8 increased (Figure 7).

## 4 DISCUSSION

This study showed that the M‐MDSC‐like cells express an alternative response comprised of changes in both soluble mediators and transcriptional regulators upon LPS activation. We also confirmed that the ex vivo endotoxin‐induced M‐MDSC‐like cells share functional (low TNF response) and phenotypic (low HLA‐DR expression) traits described for M‐MDSCs^9^ in clinical settings. Using this model, the tolerant state of the M‐MDSC‐like cells, in terms of a reduced TNF response, remained when challenged by different concentrations of LPS or other TLR ligands and cytokines. The global‐tolerant state of these cells toward a range of challenges indicates an altered cellular response mechanism rather than an impaired TLR4 signaling. Since characteristics of MDSCs have previously been investigated by global gene expression analysis,^12^ and the molecular events leading up to endotoxin tolerance has been studied intensively before,^57^ we solely focused on the functional effects of this reprogramming of monocytes. To our knowledge, this is the first study that explored the cytokine responses of endotoxin‐induced M‐MDSC‐like cells by screening a broad panel of inflammatory mediators.

All identified mediators, except for HGF, were significantly increased in either or both phenotypes compared with the unstimulated monocyte control. Although the normal monocyte response displayed a similar production of HGF as the unstimulated monocyte control, a significantly lowered level of HGF was found in the M‐MDSC‐like response. Increased HGF has been found in plasma during infection in both septic and nonseptic patients, where early phase septic patients displayed elevated levels of TNF and IL‐10,^24^ signifying an active inflammatory response. A previous study on primary monocytes found that stimulation with HGF leads to an enhanced migratory activity and invasiveness of monocytes,^25^ suggesting a proinflammatory role of HGF on monocytes. The other phenotypic molecule identified in M‐MDSC‐like response was CXCL5, which in cancer, acts as the chemoattractant for CXCR2‐expressing MDSCs, thus promoting MDSC recruitment and infiltration into tumors.^26^ In mice studies, CXCL5 was suggested to contribute to the resistance to bacterial infection^27^ and to decrease neutrophil influx to the lung.^28^ In our study, an increased level of CXCL5 in normal monocyte response was detected as well, but no statistical significance was found. Moreover, CXCL5 showed an early increase at 4 h post LPS stimulation in M‐MDSC‐like cells, whereas in normal monocytes, the increase could only be seen at 24 h (data not shown), indicating an early and prolonged CXCL5 response in the M‐MDSC‐like cells. Moreover, our inhibition data suggest that CXCL5 is partially regulated by PU.1 in M‐MDSC‐like cells. Taken together, the M‐MDSC‐like phenotypic response offers a functional link, where endotoxin‐induced M‐MDSC‐like cells could contribute to immunosuppression by affecting the composition of infiltrated immune cells to the site of inflammation.

Out of the 3 phenotypic mediators released by normal monocytes in response to LPS challenge (CXCL10, IL‐12p40, and CCL2), CXCL10 and IL‐12p40 were completely absent in LPS‐stimulated M‐MDSC‐like cells. CXCL10 and IL‐12p40 are both known inflammatory mediators that can recruit monocytes and promote Th1 responses.^29^, ^30^ The absence of CXCL10 and IL‐12p40 production by M‐MDSC‐like cells is consistent with studies describing a decreased inflammatory response in endotoxin‐tolerant cells.^31^, ^32^, ^33^ Unlike CXCL10 and IL‐12p40, CCL2 showed a slight increase also in M‐MDSC‐like cells but with no statistical significance detected. It is known that in infection, CCL2 recruits monocytes and enhances phagocytic properties.^34^ However, it has also been shown that CCL2 can attract MDSCs to the site of cancer^35^ and may induce reprogramming of monocytes into MDSCs.^36^, ^37^ This indicates that CCL2 could take part in both phenotypic responses exerting different downstream effect.

IL‐10 is known for its potent anti‐inflammatory and immunosuppressive functions^38^, ^39^, ^40^ and is a cytokine previously associated with MDSCs.^40^ In our model, IL‐10 appeared as part of the shared response, in which a higher level was seen in normal response compared with M‐MDSC‐like cell response. Although IL‐10 contributes to the development of MDSCs, it can also be produced by MDSCs.^40^, ^41^ In the current study, the normal monocytes produce significantly more IL‐10 compared with the M‐MDSC‐like cells at 24 h. However, at 24 h, the IL‐10 to TNF ratio was significantly higher in M‐MDSC‐like cells. A high TNF to IL‐10 ratio has been reversely correlated to infection outcome in severe burn patients.^42^ In addition, at earlier timepoints, M‐MDSC‐like cells showed a faster IL‐10 response after stimulation (data not shown), suggesting that altered time kinetics of IL‐10 is a characteristic of the M‐MDSC‐like cell response. A low but persistent level of IL‐10 secretion may thus be one of the mechanisms to maintain immunosuppression.

It should be noted that the 23 inflammatory modulators identified using PEA were based on a small sample size (*n* = 4). As there were big variations among the individual donors in the level of these modulators, we did further validation experiments using absolute quantitative approaches in 5 new experiments. Although some of the mediators in the validation experiments could no longer be considered specific for 1 phenotype using a more stringent statistical approach, none of them moved from one phenotype to the other.

The regulation of response to LPS in M‐MDSC‐like cells is likely to be governed by a repertoire of multiple factors, including multiple transcription factors, epigenetic regulation,^43^ and combinations thereof. In the present study, 5 candidate transcription factors were identified, and M‐MDSC‐like cells showed increased gene expression of *SPI1*, whereas the expression of *EPAS1* was suppressed. This pattern could be confirmed on the protein level for PU.1, encoded by *SPI1*, but not for *EPAS1. EPAS1*, also known as *HIF‐2α*, together with *HIF1α*, are master transcription regulators in hypoxic response.^44^ Previous studies on the expression of EPAS1 in sepsis are contradictory as both up‐regulation and suppression have been found in sepsis patients.^45^, ^46^ The difference in *EPAS1* expression between these studies may be due to the different time point of sample collection as well as the heterogeneity of septic patients.^47^

The transcription factor hSIRT1, which is encoded by *SIRT1*, showed a small but significant increase only in protein level in M‐MDSC‐like cells at 24 h after LPS challenge, whereas there was no significant difference observed in gene expression level. Gene expression analysis of *SIRT1* was performed 2 h post‐LPS, which might not be the optimal time window for detecting changes in *SIRT1*. hSIRT1 is a NAD^+^‐dependent protein deacetylase that plays a role in metabolism, inflammation, and other important physiologic as well as disease conditions.^48^ In consistent with our findings, it has been reported, in a study using RAW264.7 cells as well as primary intraperitoneal mouse macrophages, that hSIRT1 activation can suppress the release of TNF after LPS stimulation.^49^ However, it should be noted that the protein levels of the transcription factors were detected by a global proteomics method, LC–MS, which has lower sensitivity in terms of targeted protein abundance, and the differences we observed between the phenotypes were approximate 10%. Hence, the relevance of hSIRT1 in the phenotypic response of the M‐MDSC‐like cells needs to be further validated.

Last, PU.1, the transcription factor encoded by *SPI1*, and that had differential expression on both gene and protein level in our study among the 2 phenotypes, is a transcription factor of the E26 transformation‐specific family that is involved in both early development of immune cells and mature immune cells function.^43^, ^50^ PU.1 was shown as one of the key transcription regulators of TLR 4^51^ as well as for several genes of cytokines, chemokines, and CSFs,^52^ such as *TNF* in mouse dendritic cells and *IL12B* in human monocytes. However, in our study, inhibition of PU.1 did not change the level of IL‐12p40 and TNF in the M‐MDSC‐like cells, indicating possible differential regulations in the 2 phenotypes. Our finding of an increased expression of both *SPI1* and PU.1 in M‐MDSC‐like cells is in line with a study in which up‐regulation and demethylation of *SPI1* has been shown in postseptic immunosuppressed monocytes.^53^ PU.1 has been suggested to be involved in the regulation of IL‐10 expression,^54^ and there was an observed effect of PU.1 inhibition on IL‐10 production in the current study. However, no significant differences could be observed to the DMSO control, suggesting the major effect on IL‐10 might be due to nonspecific effects of DMSO. In addition to the effects on CXCL5 mentioned above, we also observed an increase in CCL8 in M‐MDSC‐like during PU.1 inhibition (with levels comparable to normal monocytes). CCL8, also known as MCP‐2, plays an important role in recruiting a large number of different immune cells in inflammatory conditions^55^ and has also been shown to attracting tumor‐associated macrophages and promote metastasis in cancer.^56^ In addition, increased CCL8 levels have also been shown in septic patients’ plasma compared with healthy volunteers at the early stages of sepsis.^57^ However, the levels of circulating CCL8 levels during later stages, when immunosuppression is more pronounced, remain unknown.

In conclusion, we have identified a profile comprised of mediators that sets M‐MDSC‐like cell responses apart from that of the normal monocyte following threat recognition (LPS response). These mediators could be traced back to a group of 3 potential transcription factors with differential expression in M‐MDSC‐like cells, where particularly PU.1 displayed consistent patterns of up‐regulation in M‐MDSC‐like cells. PU.1 in turn regulated the production of the chemokines CXCL5 and CCL8, thus affecting the ability of M‐MDSC‐like cells to recruit other immune cells. However, additional studies are needed to further investigate the role of the identified mediators in the development of a persistent immunosuppression. Also, as these findings are limited to in vitro, the mediators should be evaluated in a clinical setting of immunosuppressive septic patients, to further complement the knowledge of how reprogrammed monocytes controls and regulates the immune response and contributes to excessive immunosuppression in systemic inflammatory diseases.

## DISCLOSURE

The authors declare no conflict of interest.

## AUTHORSHIP

K. T. and K. M. performed the experiments, analyzed the data, interpreted the results, and wrote the manuscript. E. V. and R. Ka. performed data curation, formal analysis, and reviewed and edited the manuscript. E. S. designed the study, interpreted the results, reviewed, and edited the manuscript. A. P. designed the study, analyzed the data, interpreted the results, reviewed, and edited the manuscript. R. Kr. interpreted the results, reviewed, and edited the manuscript. D. E. designed the study, performed the experiments, analyzed the data, interpreted the results, reviewed, and edited the manuscript. K. T. and K. M. contributed equally to this work.

## Supporting information

Supplemental Figure 1. TNF response in M‐MDSC‐like cells upon stimulation with other inflammatory agonists.

Supplementary Information

Supplementary materials

Supplementary Information
