# Supplementary figures and images for: Cytokine responses to LPS in reprogrammed monocytes are associated with the transcription factor PU.1
Source: J Leukoc Biol. 2022 Mar 13;112(4):679–92. doi: 10.1002/JLB.3A0421-216R (PMC9790682; doi:10.1002/JLB.3A0421-216R)

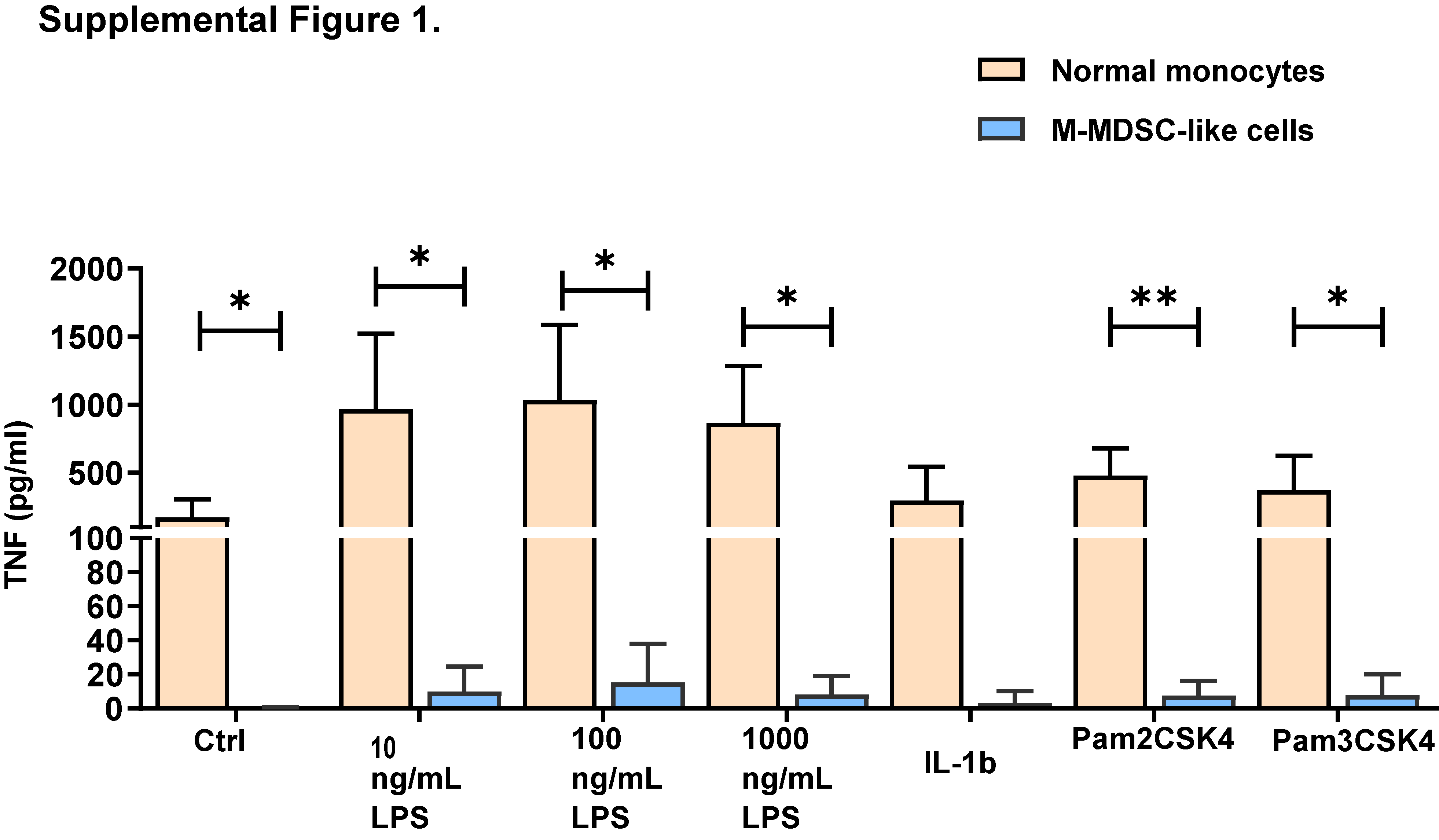

Supplement: Supplementary file 1 — Supplemental Figure 1. TNF response in M‐MDSC‐like cells upon stimulation with other inflammatory agonists. [file JLB-112-679-s001.tif]
