# Supplementary material for: Cytokine responses to LPS in reprogrammed monocytes are associated with the transcription factor PU.1
Source: J Leukoc Biol. 2022 Mar 13;112(4):679–92. doi: 10.1002/JLB.3A0421-216R (PMC9790682; doi:10.1002/JLB.3A0421-216R)
Supplement: Supplementary file 2 — Supplementary Information [file JLB-112-679-s003.docx]

**Supplemental Figure 1. TNF response in M-MDSC-like cells upon stimulation with other inflammatory agonists.** Monocytes (normal monocytes) and M-MDSC-like cells (M-MDSC-like cells) were challenged with either recombinant human IL-1β, Pam2CSK4 (synthetic diacylated lipoprotein), Pam3CSK4 (synthetic triacylated lipopeptide) or different concentration of LPS. TNF levels were determined at 24h (n=5). Statistical analysis was performed using paired t test. Data are log transformed, shown as mean and standard deviation. Values of p < 0.05 (*), p < 0.01(**), p < 0.001(***) were considered significant.
