# Supplementary material for: Cytokine responses to LPS in reprogrammed monocytes are associated with the transcription factor PU.1
Source: J Leukoc Biol. 2022 Mar 13;112(4):679–92. doi: 10.1002/JLB.3A0421-216R (PMC9790682; doi:10.1002/JLB.3A0421-216R)
Supplement: Supplementary file 3 — Supplementary materials [file JLB-112-679-s004.docx]

### Sample preparation for quantitative proteomics analysis

24 h after LPS challenge, 1x10^6^ cells from each phenotype were pelleted and flash-frozen in liquid nitrogen before stored in -80 °C. Samples were homogenized using lysis matrix D (1.4 mm ceramic spheres) on a FastPrep®-24 instrument (MP Biomedicals, Irvine, CA) for 5 repeated 40 sec cycles at 6.5 m/s in the lysis buffer containing 2 % sodium dodecyl sulfate (SDS) and 50 mM triethylammonium bicarbonate (TEAB). Lysed samples were centrifuged at 21 100 x g for 10 min and the supernatants were transferred to clean tubes. Protein concentrations were determined using Pierce™ BCA Protein Assay Kit (Thermo Fischer Scientific) and the Benchmark™ Plus microplate reader (Bio-Rad Laboratories, Hercules, CA) with bovine serum albumin solutions as standards.

Aliquots containing 30 µg of total protein from each sample were incubated at 37°C for 60 min in the lysis buffer with DL-dithiothreitol at 100 mM final concentration. The reduced samples were processed using the modified filter-aided sample preparation (FASP) method [1]. In short, the reduced samples were diluted to 1:4 by 8 M urea solution, transferred onto Nanosep 30k Omega filters (Pall Corporation, Port Washington, NY) and washed repeatedly with 8 M urea and once with digestion buffer (0.5% sodium deoxycholate in 50 mM triethylammonium bicarbonate). Free cysteine residues were modified using 10 mM methyl methanethiosulfonate solution in digestion buffer for 20 min at room temperature and the filters were washed twice with 100 µL of digestion buffer. Pierce trypsin protease (MS Grade, Thermo Fisher Scientific) in digestion buffer was added at a ratio of 1:100 relative to total protein mass and the samples were incubated at 37°C overnight. An additional portion of trypsin was added and incubated for another two hours.

The peptides were collected by centrifugation and labelled using Tandem Mass Tag (TMT 10plex) reagents (Thermo Fischer Scientific) according to the manufacturer’s instructions. The labelled samples were combined into one pooled sample, concentrated using vacuum centrifugation, and sodium deoxycholate was removed by acidification with 10% TFA and subsequent centrifugation. The labelled pooled sample was treated with Pierce peptide desalting spin columns (Thermo Fischer Scientific) according to the manufacturer’s instructions.

The combined samples were fractionated into 40 primary fractions by basic-pH reversed-phase chromatography (bRP-LC) using a Dionex Ultimate 3000 UPLC system (Thermo Fischer Scientific). Peptide separations were performed on a reversed-phase XBridge BEH C18 column (3.5 μm, 3.0x150 mm, Waters Corporation, Milford, MA) using a linear gradient from 3 to 40 % solvent B over 18 min followed by an increase to 100 % B over 5 min and hold at 100 % B for 5 min. Solvent A was 10 mM ammonium formate buffer at pH 10.00 and solvent B was 90 % acetonitrile, 10 % 10 mM ammonium formate at pH 10.00. The primary fractions were concatenated into final 20 fractions (1+21, 2+22, … 20+40), evaporated and reconstituted in 15 μL of 3 % acetonitrile, 0.2 % formic acid for LC-MS analysis.

### Liquid chromatography-mass spectrometry (LC-MS)

The fractions were analyzed on an Orbitrap Fusion Lumos Tribrid mass spectrometer interfaced with an Easy-nLC 1200 liquid chromatography system (both Thermo Fisher Scientific). Peptides were trapped on an Acclaim Pepmap 100 C18 trap column (100 μm x 2 cm, particle size 5 μm, Thermo Fischer Scientific) and separated on an analytical column (75 μm x 35 cm, packed in-house with Reprosil-Pur C18, particle size 3 μm, Dr. Maisch, Ammerbuch, Germany) using a linear gradient from 5 % to 33 % B over 77 min followed by an increase to 100 % B for 3 min, and 100 % B for 10 min at a flow of 300 nL/min. Solvent A was 0.2 % formic acid in water and solvent B was 80 % acetonitrile, 0.2 % formic acid.

MS scans were performed at 120 000 resolution in the m/z range 375-1375. The most abundant doubly or multiply charged precursors from the MS1 scans were isolated using the quadrupole with 0.7 m/z isolation window with a “top speed” duty cycle of 3 sec and dynamic exclusion within 10 ppm for 45 sec. The isolated precursors were fragmented by collision induced dissociation (CID) at 35 % collision energy with the maximum injection time of 50 msec and detected in the ion trap, followed by multinotch (simultaneous) isolation of the top 10 MS2 fragment ions within the m/z range 400-1400, fragmentation (MS3) by higher-energy collision dissociation (HCD) at 65 % collision energy and detection in the Orbitrap at 50 000 resolution, m/z range 100-500 and maximum injection time 105 msec.

The abovementioned samples were re-injected and MS scans were performed at 120 000 resolution in two m/z ranges: 470-610 and 600-1250. Targeted inclusion list was prepared for the selected peptides of the proteins P25963 (NF-kappa-B inhibitor alpha), Q16665 (Hypoxia-inducible factor 1-alpha, HIF1-alpha) and Q99814 (Endothelial PAS domain-containing protein 1, EPAS-1) that were detectable according the data in ProteomicsDB (<https://www.ProteomicsDB.org>) [2]. Precursor ions with the correct charge and the monoisotopic mass within 15 ppm of the theoretical mass in the inclusion list were fragmented by collision induced dissociation at 35 % collision energy with the maximum injection time of 150 ms and detected in the Orbitrap at 30 000 resolution, followed by multinotch (simultaneous) isolation of the top 10 MS2 fragment ions within the m/z range 400-1400, fragmentation (MS3) by higher-energy collision dissociation (HCD) at 65 % collision energy and detection in the Orbitrap at 50 000 resolution, m/z range 100-500 and maximum injection time 200 msec.

### Proteomic Data Analysis

Proteins were identified and quantified using Proteome Discoverer version 2.4 (Thermo Fisher Scientific). The database matching was performed using the Mascot search engine v. 2.5.1 (Matrix Science, London, UK) against the Swiss-Prot *Homo sapiens* database. Trypsin was used as a cleavage rule with no allowed missed cleavages; methylthiolation on cysteine residues, TMT at peptide N-termini and on lysine side chains were set as static modifications, and oxidation on methionine was set as a dynamic modification. Precursor mass tolerance was set to 5 ppm and fragment ion tolerance to 0.6 Da. Percolator was used for peptide-spectrum match (PSM) validation with the strict false discovery rate (FDR) threshold of 1 %. The TMT reporter ions were identified with 3 mmu mass tolerance in the MS3 HCD spectra and the TMT reporter S/N values for each sample were normalized within Proteome Discoverer 2.4 on the total peptide amount. Only the unique identified peptides were taken into account for protein quantification. The LC-MS files from the re-injection experiment were processed largely via the same workflow with the following changes: protein database consisted only of the proteins P25963 (NF-kappa-B inhibitor alpha), Q16665 (HIF1-alpha) and Q99814 (EPAS-1); precursor mass tolerance was set to 10 ppm and the fragment ion tolerance in MS2 to 0.03 Da; Fixed Value PSM Validator was used instead of Percolator.

References:

1. Wiśniewski JR, Zougman A, Nagaraj N, et al. Universal sample preparation method for proteome analysis. *Nat Methods*. 2009;6:359–362
2. Samaras P, Schmidt T, Frejno M, et al. ProteomicsDB: a multi-omics and multi-organism resource for life science research. *Nucleic Acids Res*. 2020;48:D1153–D1163.
