# Supplementary material for: Cytokine responses to LPS in reprogrammed monocytes are associated with the transcription factor PU.1
Source: J Leukoc Biol. 2022 Mar 13;112(4):679–92. doi: 10.1002/JLB.3A0421-216R (PMC9790682; doi:10.1002/JLB.3A0421-216R)
Supplement: Supplementary file 4 — Supplementary Information [file JLB-112-679-s002.docx]

**The Exploring Inflammation in Health and Disease (X-HiDE) Consortium members**

The Exploring Inflammation in Health and Disease (X-HiDE) Consortium is a research consortium within the field of inflammation based at Örebro university. More information can be found at: <https://www.oru.se/x-hide/>.

| **Name** | **Academic title** | **Affiliation(s)** |
| --- | --- | --- |
| Kedeye Tuerxun^1,2^ | MSc | ^1^School of Medical Sciences, Faculty of Medicine and Health, Örebro University, 701 82 Örebro, Sweden.  ^2^Inflammatory Response and Infection Susceptibility Centre (iRiSC), Faculty of Medicine and Health, Örebro University, 701 82 Örebro, Sweden. |
| Eva Särndahl^1,2^ | PhD, Prof | ^1^School of Medical Sciences, Faculty of Medicine and Health, Örebro University, 701 82 Örebro, Sweden.  ^2^Inflammatory Response and Infection Susceptibility Centre (iRiSC), Faculty of Medicine and Health, Örebro University, 701 82 Örebro, Sweden. |
| Alexander Persson^1,2^ | PhD | ^1^School of Medical Sciences, Faculty of Medicine and Health, Örebro University, 701 82 Örebro, Sweden.  ^2^Inflammatory Response and Infection Susceptibility Centre (iRiSC), Faculty of Medicine and Health, Örebro University, 701 82 Örebro, Sweden. |
| Robert Kruse^1,2,3^ | PhD | ^1^School of Medical Sciences, Faculty of Medicine and Health, Örebro University, 701 82 Örebro, Sweden.  ^2^Inflammatory Response and Infection Susceptibility Centre (iRiSC), Faculty of Medicine and Health, Örebro University, 701 82 Örebro, Sweden.  ^3^Department of Clinical Research Laboratory, Faculty of Medicine and Health, Örebro University, 701 82 Örebro, Sweden. |
| Daniel Eklund^1,2^ | PhD | ^1^School of Medical Sciences, Faculty of Medicine and Health, Örebro University, 701 82 Örebro, Sweden.  ^2^Inflammatory Response and Infection Susceptibility Centre (iRiSC), Faculty of Medicine and Health, Örebro University, 701 82 Örebro, Sweden. |
| Niloofar Nickaeen^1^ | PhD | ^1^School of Medical Sciences, Faculty of Medicine and Health, Örebro University, 701 82 Örebro, Sweden. |
| Alexander Hedbrant^1,2^ | PhD | ^1^School of Medical Sciences, Faculty of Medicine and Health, Örebro University, 701 82 Örebro, Sweden.  ^2^Inflammatory Response and Infection Susceptibility Centre (iRiSC), Faculty of Medicine and Health, Örebro University, 701 82 Örebro, Sweden. |
| Ignacio Rangel^1,2^ | PhD | ^1^School of Medical Sciences, Faculty of Medicine and Health, Örebro University, 701 82 Örebro, Sweden.  ^2^Nutrition-Gut-Brain Interactions Research Centre, Faculty of Medicine and Health, Örebro University, 701 82 Örebro, Sweden. |
| Allan Sirsjö^1,2^ | PhD, Prof | ^1^School of Medical Sciences, Faculty of Medicine and Health, Örebro University, 701 82 Örebro, Sweden.  ^2^Cardiovascular Research Centre, School of Medical Sciences, Örebro University, 701 82 Örebro, Sweden. |
| Hans Fredlund^1^ | MD, PhD | ^1^School of Medical Sciences, Faculty of Medicine and Health, Örebro University, 701 82 Örebro, Sweden. |
| Lisa Kurland^1,2^ | PhD, MD, Prof | ^1^School of Medical Sciences, Faculty of Medicine and Health, Örebro University, 701 82 Örebro, Sweden.  ^2^Inflammatory Response and Infection Susceptibility Centre (iRiSC), Faculty of Medicine and Health, Örebro University, 701 82 Örebro, Sweden. |
| Ioannis Parodis^1,2^ | MD, PhD | ^1^Division of Rheumatology, Department of Medicine Solna, Karolinska Institutet and Karolinska University Hospital, 171 77 Stockholm, Sweden  ^2^Department of Rheumatology, Faculty of Medicine and Health, Örebro University, 701 82 Örebro, Sweden |
| Eewa Nånberg^1,2^ | PhD, Prof | ^1^School of Health Sciences, Faculty of Medicine and Health, Örebro University, 701 82 Örebro, Sweden.  ^2^Inflammatory Response and Infection Susceptibility Centre (iRiSC), Faculty of Medicine and Health, Örebro University, 701 82 Örebro, Sweden. |
| Klemen Camernik^1^ | PhD | ^1^School of Medical Sciences, Faculty of Medicine and Health, Örebro University, 701 82 Örebro, Sweden. |
| Katarina Persson^1,2^ | PhD, Prof | ^1^School of Medical Sciences, Faculty of Medicine and Health, Örebro University, 701 82 Örebro, Sweden.  ^2^Inflammatory Response and Infection Susceptibility Centre (iRiSC), Faculty of Medicine and Health, Örebro University, 701 82 Örebro, Sweden. |
| Dirk Repsilber^1^ | PhD, Prof | ^1^School of Medical Sciences, Faculty of Medicine and Health, Örebro University, 701 82 Örebro, Sweden. |
| Antje Thonig^1^ |  | ^1^School of Medical Sciences, Faculty of Medicine and Health, Örebro University, 701 82 Örebro, Sweden. |
| Anette Oskarsson^1^ |  | ^1^Grants Office, Örebro University, 701 82 Örebro, Sweden. |
| Samira Salihovic^1,2^ | PhD | ^1^School of Medical Sciences, Faculty of Medicine and Health, Örebro University, 701 82 Örebro, Sweden.  ^2^Inflammatory Response and Infection Susceptibility Centre (iRiSC), Faculty of Medicine and Health, Örebro University, 701 82 Örebro, Sweden. |
| Matej Orešič^1,2^ | PhD, Prof | ^1^School of Medical Sciences, Faculty of Medicine and Health, Örebro University, 701 82 Örebro, Sweden.  ^2^Turku Bioscience Centre, University of Turku and Åbo Akademi University, 205 20 Turku, Finland. |
| Simon Athlin^1,2^ | MD, PhD | ^1^School of Medical Sciences, Faculty of Medicine and Health, Örebro University, 701 82 Örebro, Sweden.  ^2^Inflammatory Response and Infection Susceptibility Centre (iRiSC), Faculty of Medicine and Health, Örebro University, 701 82 Örebro, Sweden |
| Jacob Ehnfors^1^ | PhD | ^1^Department of Communication and Collaboration, Örebro University, 701 82 Örebro, Sweden |
| Heather Marshall-Heyman^1^ | PhD | ^1^InnoFund innovation funding advisors AB, 182 38 Danderyd, Sweden |
| Sofia Lykkeklev^1^ |  | ^1^School of Medical Sciences, Faculty of Medicine and Health, Örebro University, 701 82 Örebro, Sweden. |
| Mikael Kubista^1,2^ | PhD | ^1^Laboratory of Gene Expression, Institute of Biotechnology CAS, BIOCEV, 252 50 Vestec, Czech Republic  ^2^TATAA Biocenter AB, 412 51 Gothenburg, Sweden. |
| Martin Andersson^1^ | PhD | ^1^Sprint Bioscience, 141 57 Huddinge, Sweden |
| Gunnar Cedersund^1^ | PhD | ^1^Department of Biomedical Engineering, Linköping University, 581 83 Linköping, Sweden |
| Elin Nyman^1^ | PhD | ^1^Department of Biomedical Engineering, Linköping University, 581 83 Linköping, Sweden |
| Olaf Wolkenhauer^1^ | PhD, Prof | University of Rostock, Department of Systems Biology, 180 51 Rostock, Germany |
| Shailendra Gupta^1^ | PhD | ^1^University of Rostock, Department of Systems Biology, 180 51 Rostock, Germany |
| Joachim Almquist^1^ | PhD | ^1^Clinical Pharmacology and Quantitative Pharmacology, Clinical Pharmacology & Safety Sciences, R&D, AstraZeneca, 431 50 Gothenburg, Sweden |
| Jan Brugård^1^ | MSc | ^1^Wolfram MathCore AB, 583 30 Linköping, Sweden |
